# 

**Published:** 1891-10

**Authors:** 


Entered at the Post Office at Austin, Texas, as Second-Class Mail Matter.

VOL. VII. OCTOBER, 1891. No. 4.

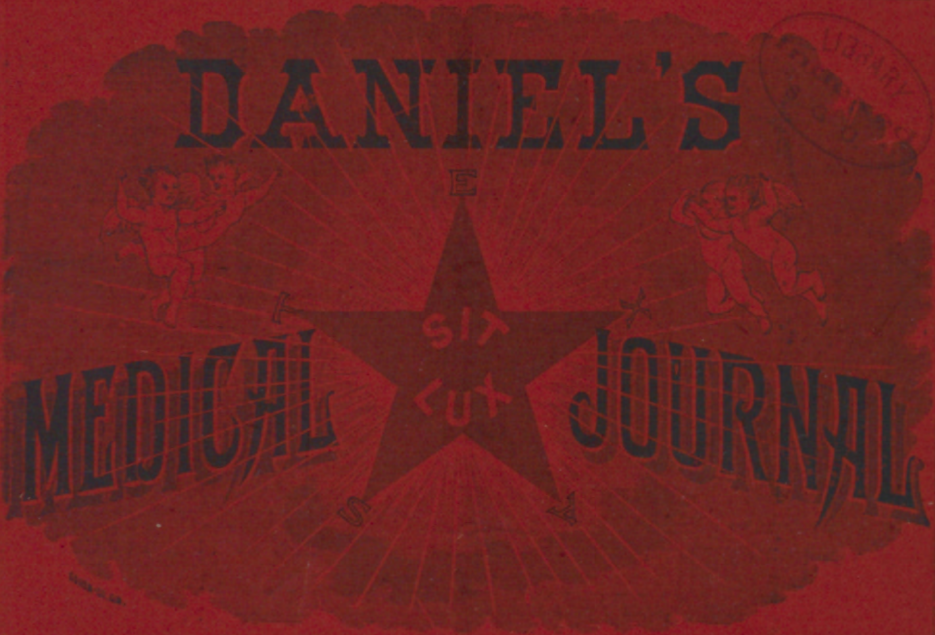

### DANIEL'S MEDICAL JOURNAL

Subscription, $2.00 a Year, in Advance.— Single Copies, Twenty-five Cents.

F. E. DANIEL, M. D., Editor and Publisher, AUSTIN, TEXAS.

